# Comparison of the effects of BDNF/TRKB signalling on metabolic biomarkers in the liver of sedentary and trained rats with normal and knockout BDNF genotypes

**DOI:** 10.3389/fphys.2023.1268648

**Published:** 2023-12-13

**Authors:** Norbert Grzelak, Dominik Kaczmarek, Włodzimierz Mrówczyński

**Affiliations:** ^1^ Department of Neurobiology, Poznań University of Physical Education, Poznań, Poland; ^2^ Department of Physiology and Biochemistry, Poznań University of Physical Education, Poznań, Poland

**Keywords:** BDNF, knockout genotype, endurance training, liver, rats

## Abstract

**Introduction:** The effect of brain-derived neurotrophic factor (BDNF) on the modulation of metabolic processes in the liver is poorly understood. Therefore, the aim of this study was to investigate whether hepatic concentrations or activities of metabolic biomarkers depend on altered BDNF/TrkB content in the liver, resulting from different BDNF genotypes of rats. In addition, it was assessed whether 5-week moderate endurance training modifies the levels of BDNF/Trk-B signaling and studied hepatic markers.

**Methods:** Experiments were performed on wild-type and heterozygous BDNF knockout (HET, SD-Bdnf) rats, which were divided into four groups: control with normal genotype (Bdnf+/+), control with BDNF knockout genotype (Bdnf+/−), trained with normal genotype (Bdnf+/+T) and trained with BDNF knockout genotype (Bdnf +/−T). BDNF/TrkB concentrations as well as selected metabolic biomarkers including lipids—total cholesterol (CHOL), low-density lipoprotein (LDL), triglycerides (TG); enzymes—alanine aminotransferase (ALAT), aspartate aminotransferase (ASAT), gamma-glutamyl transferase (GGT), lactate dehydrogenase (LDH), alkaline phosphatase (ALP); hormones—insulin (INS) and leptin (LEPT) as well as interleukin-6 (IL-6) as regeneration indicator were measured directly in liver homogenates.

**Results and Discussion:** The study showed that Bdnf+/− rats exhibited reduced BDNF/TrkB signaling (BDNF, *p* < 0.0001; Trk-B, *p* = 0.0005), altered lipid levels (CHOL, *p* < 0.0001; LDL, *p* < 0.0001; TG, *p* = 0.0006) and reduced hepatic ALAT (*p* = 0.0004) and GGT (*p* < 0.0001) activity, which may contribute to hepatic steatosis and obesity, as well as indicate impairment of specific metabolic pathways in the liver. Interestingly, endurance training did not alter hepatic BDNF and TrkB content, but improved ALAT (*p* = 0.0366) and ASAT (*p* = 0.0191) activities and increased hepatic IL-6 (*p* = 0.0422) levels in Bdnf +/− rats, suggesting enhanced liver regeneration in animals with BDNF allele loss.

## Introduction

Brain-derived neurotrophic factor (BDNF) is a protein involved in the modulation of numerous biological processes occurring in the central and peripheral nervous systems in mammals (Li et al., 2022). BDNF has been shown to have the ability to activate transmembrane tyrosine kinase B (Trk-B) receptors in neurons (Guo et al., 2014; Jin, 2020), which triggers various intracellular signaling pathways to alter specific neuronal functions (Yoshii and Constantine-Paton, 2010). However, BDNF and its high-affinity receptors have also been found in skeletal (Mousavi and Jasmin, 2006) and smooth muscles (Aravamudan et al., 2016), the endocrine system (Podyma et al., 2021), adipose tissue (Nakagomi et al., 2015) as well as some inner organs such as the heart (Pius-Sadowska and Machaliński, 2017), pancreas (Fulgenzi et al., 2020) and liver (Genzer et al., 2017), which plays a vital role in many biological processes, including metabolism (Han et al., 2016), detoxification (Grant, 1991), and the production of various hormones and proteins (Canbay et al., 2007).

In the liver, the sources of several neurotrophins and their receptors (including BDNF and Trk-B) are hepatic stellate cells (Cassiman et al., 2001) and hepatocytes (Genzer et al., 2017), which are the main cell types in this organ. The hepatic BDNF/Trk-B axis has been shown to play a key role in maintaining liver innervation and haematopoietic function (García-Suárez et al., 2006) and biliary remodelling during cholestasis (Vivacqua et al., 2014). In addition, it has been shown that hepatic BDNF levels can be useful for diagnosing liver function in patients with cirrhosis (Shu et al., 2019), and associated with liver fibrosis in alcohol use disorders (Girard et al., 2021). Studies in rodents and humans (Chaldakov et al., 2004; Motamedi et al., 2017) have shown that low levels of circulating BDNF are associated with metabolic dysfunctions such as obesity, metabolic syndrome and related disorders (e.g., diabetes, cardiovascular disease). Experiments on mice lacking the gene encoding BDNF (Lyons et al., 1999; Kernie et al., 2000) have shown that exogenous administration of BDNF can reverse the effects of metabolic syndrome. Furthermore, Genzer et al. (2017) showed that BDNF binding to specific receptors in rat liver hepatocytes activates catabolic pathways, such as fatty acid oxidation, in parallel with inhibition of gluconeogenesis and increased glycogen storage. All these reports indicate an important regulatory role for BDNF/TrkB axis in liver tissue (Hang et al., 2021).

It is well known that physical activity, especially of endurance nature, can modulate serum/plasma concentration of BDNF (Szuhany et al., 2015; Dinoff et al., 2016). The results of most human studies suggest considerable elevation of BDNF concentration in blood serum after acute endurance training lasting several weeks (Tang et al., 2008; Rasmussen et al., 2009; Arazi et al., 2021; Ribeiro et al., 2021). Moreover, Belviranlı and Okudan (2018) showed that voluntary wheel training in rats increased not only BDNF levels in plasma but also in liver tissue. In addition, another study in rats showed that the application of moderate to high-intensity training leads to both increased plasma BDNF levels and insulin tolerance, which are suppressed by daily intraperitoneal administration of the Trk-B blocker (Jiménez-Maldonado et al., 2014).

Hence, existing evidence suggests that there may be a strong association between BDNF/Trk-B axis and metabolic products in the liver and physical activity. The purpose of this study was to investigate the potential link between changes in the hepatic BDNF/TrkB axis induced by different BDNF genotype (normal vs. knockout rats) and endurance training (trained vs. untrained rats) and the activity and concentration of metabolic biomarkers in the liver. Knockout rats were selected due to their reduced serum BDNF levels ranging from 20% (Grzelak et al., 2023) to 73% (Harris et al., 2016), while 5-week endurance training was used to increase BDNF levels in the rat liver.

For this reason, two types of rats were used in this study: wild-type and BDNF heterozygous knockout (HET, SD-Bdnf), some of which were subjected to an endurance training procedure. The following four groups of rats were studied in the experiments conducted: 1) with typical hepatic BDNF levels (control with a normal genotype, Bdnf+/+), 2) with altered hepatic BDNF levels [control with BDNF knockout genotype (HET, SD-BDNF, Bdnf+/−)], and (3, 4) trained with normal (Bdnf+/+T) and knockout genotype (Bdnf+/-T) that underwent 5-week endurance training procedure.

In liver homogenates of these rats levels of BDNF, Trk-B as well as concentration/activity of some lipids: total cholesterol (CHOL), low-density lipoprotein (LDL) and triglycerides (TG), enzymes: lactate dehydrogenase (LDH), alanine aminotransferase (ALAT), aspartate aminotransferase (ASAT), gamma-glutamyl transferase (GGT) and alkaline phosphatase (ALP) and hormones: insulin (INS) and leptin (LEPT) were measured. In addition, levels of hepatic interleukin-6 (IL-6) which is an essential factor indicating liver homeostasis, regeneration, infection defence and fine tuning of metabolic functions (Schmidt-Arras and Rose-John, 2016) were also measured in all four studied groups of animals.

Relationships between hepatic amounts of both BDNF/Trk-B and the aforementioned specific metabolic factors were determined separately for each group and then compared among all studied populations. We hypothesised that this approach would allow us to demonstrate how genotype and physical activity affect the association between BDNF/Trk-B axis and metabolic factors in the rat liver.

## Materials and methods

### Animals

Experiments were carried out in 41 Sprague-Dawley male rats aged 4–5 months, obtained from a breeding colony at SAGE Labs (St. Louis, MO, United States). 21 animals were wild-type and had the typical BDNF genotype (*Bdnf*-wild type), while the remaining 20 animals were heterozygotes, in which *Bdnf* was knocked out using targeted zinc finger nuclease technology (*Bdnf-*knockout, SD-BDNF). The respective liver biomarkers were measured separately in rats from four different randomly selected groups: control normal genotype rats *(Bdnf*+/+, *n* = 11, average weight before experiment—524 g), control knockout genotype rats (*Bdnf*+/−, *n* = 11, average weight before experiment—681 g), trained normal genotype rats (*Bdnf*+/+T, *n* = 10, average weight before experiment—484 g) and trained knockout rats (*Bdnf*+/−T, *n* = 9, average weight before experiment—588 g). All animals were kept in standard laboratory cages (2 rats of the same genotype per cage) with *ad libitum* access to food and water. The room in which the animals were housed had controlled environmental conditions (a reverse 12 h:12 h light/dark cycle, 55% ± 1% humidity, and 22°C ± 2°C). The animals were euthanized with an overdose of sodium pentobarbital (180 mg kg^−1^) prior to liver collection. The experiments were conducted in accordance with the Polish Animal Protection Act, European Union regulations and ARRIVE guidelines. The experimental procedures were approved by the Local Ethics Committee for Animal Research in Poznań (approval number 58/2018).

### Endurance training protocol

In order to examine the effect of endurance training on BDNF/Trk-B signaling and metabolic biomarkers in the liver, moderate intensity training on an electric treadmill for small rodents (Exer-6M, Columbus Instruments) was used in groups of *Bdnf*+/+T and *Bdnf*+/-T rats. The training regimen consisted of 1 week of adaptation and 5 weeks of core training (45 min per day, 5 days per week of continuous running with gradually increasing speed up to 25 m/min at the last week of the training session (Grzelak et al., 2023).

### Liver collection and homogenization

After anaesthesia, livers of experimental animals were collected, weighed and then placed in cryogenic vials (Yancheng Huida Medical Instruments, China), which were frozen by immersion in liquid nitrogen at–80°C and stored for further measurements. Small distal portions of each lobe of the collected livers were cut off for homogenization. The obtained tissue sections were weighed, and depending on their weight, an appropriate amount (at a ratio of 1:9) of Phosphate Buffered Saline (PBS) with Halt Protease Inhibitor Cocktail EDTA-free (100X) (Thermo Fischer Scientific, United States) was added. Tissue samples were homogenised with the prepared buffer using a dispersion homogenizer (VWR VDI 12, Singapore) in four cycles (each 30 s, with 1 min cooling in ice between cycles) to 30.000 revolutions per minute (rpm). The homogenate was centrifuged at 5,000 rpm by 5 min and the obtained supernatant was kept at—80°C.

### Enzyme-linked immunosorbent assay

Levels of BDNF (sensitivity test: 0.035 ng ml^−1^, cat. number: SRB-T-81493), TrkB (sensitivity test: 12.337 pg/mL; cat. number: 201-11-0426), LEPT (sensitivity test: 7.054 pg/mL; cat number: 201-11-0456), IL-6 (sensitivity test: 1.822 pg/mL; cat number: 201-11-0136) and activity of INS (sensitivity test: 0.102 mIU/L; cat number: 201-11-0708) were determined in liver by commercial ELISA kits according to descriptions of producent (Sunredbio, China). A multi-mode microplate reader (Synergy 2 SIAFRT, BioTek, United States) was used for read of absorbance at 450 nm.

### Biochemistry analyses

Levels of LDL (sensitivity test: 3.9 mg/dL; cat. number: 7–280 [Cormay, Poland]), CHOL (sensitivity test: 1.95 mg/dL; cat. number: 7–204 [Cormay, Poland]), TG (sensitivity test: 1.4 mg/dL; cat. number 7–253 [Cormay, Poland]), activities of ALAT (sensitivity test: 8 U/L; cat. number 7–216 [Cormay, Poland]), ASAT (sensitivity test: 7 U/L; cat. number 7–214 [Cormay, Poland]), LDH (sensitivity test: 6.6 U/L; cat number. 7–239 [Cormay, Poland]), GGT (sensitivity test: 8 U/L; cat number 7–224, [Cormay, Poland]), and ALP (sensitivity test: 7.8 U/L; cat number 7–212 [Cormay, Poland]) were measured with clinical chemistry analyzer (Accent 220S, Cormay, Poland).

### Statistical analysis

Statistical analyses were carried out with Statistica 13 software (Statsoft, Poland, Cracow). Data were presented as the mean ± standard deviation (SD) for all obtained results. Two-way analysis of variance (ANOVA) was used in order to perform inter-group comparison of molecules concentrations as well as enzymes and hormone activities. Tukey’s HSD *post hoc* test was employed for determination of statistical significance between compared groups when the interaction between effects was statistically significant.

## Results

### Liver and body weights

**FIGURE 1:**
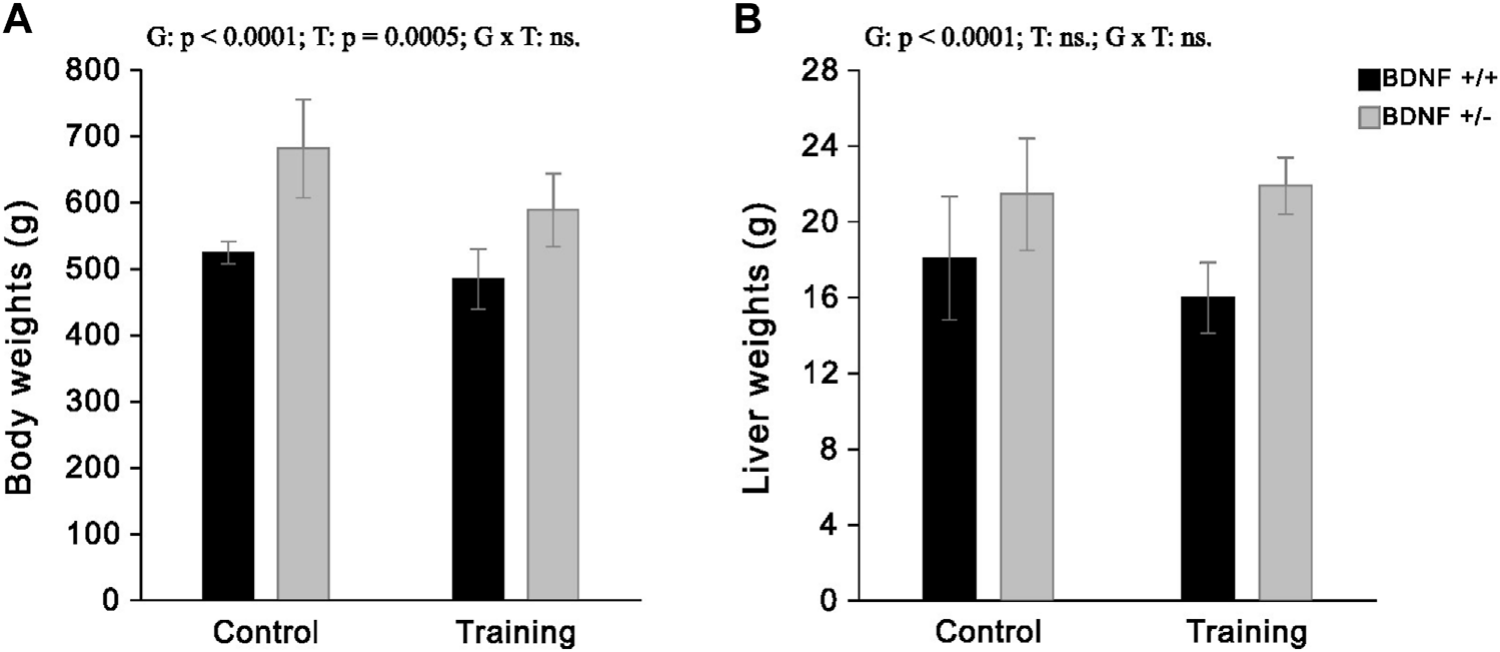
The mean values (±SD) of body weights **(A)** and liver weights **(B)** of rats from control (left side) and trained (right side) groups. *Bdnf*+/+ rats are indicated by black, while *Bdnf*+/− rats are indicated by grey. Comparisons between groups were made by two-way ANOVA. (G) genotype (*Bdnf*+/+ genotype vs. *Bdnf*+/− genotype); T—training (control vs. training); G x T—the interaction between genotype and training. For significant interactions Tukey’s *post hoc* test was performed. Significant results are denoted by p, while non-significant results are denoted by ns. Effects for body weights: Genotype—F = 56.84, *p* < 0.0001, ηp2 = 0.63; Training—F = 14.76, *p* = 0.0005, ηp2 = 0.31; Genotype x Training—F = 2.35, *p* = 0.1351, ηp2 = 0.07. Effects for liver weights: Genotype - F = 29.30, *p* < 0.0001, ηp2 = 0.47; Training—F = 0.92, *p* = 0.0005, ηp2 = 0.03; Genotype x Training - F = 2.18, *p* = 0.1494, ηp2 = 0.06.

The study showed that the average liver weight of knockout rats was 19% greater than rats with the normal genotype. However, the applied endurance training on treadmill led to a loss of liver weight only in normal genotype rats (by an average of 12%) and did not cause a reduction in the weight of this organ in the group with BDNF knockout genotype. Moreover, our previous study, performed on the same groups of animals (Grzelak et al., 2023), showed that knockout rats had a higher body mass than the normal genotype rats and also indicated that moderate-intensity running training caused weight loss in both trained normal *(Bdnf+/+T)* and knockout *(Bdnf+/*−*T)* rats. A detailed comparison of both body weight and liver weight in the four groups of rats tested (*Bdnf*+/+, *Bdnf*+/−, *Bdnf*+/+T, *Bdnf*+/−T) is shown in Figure 1.

### Genotype and training effects

**FIGURE 2:**
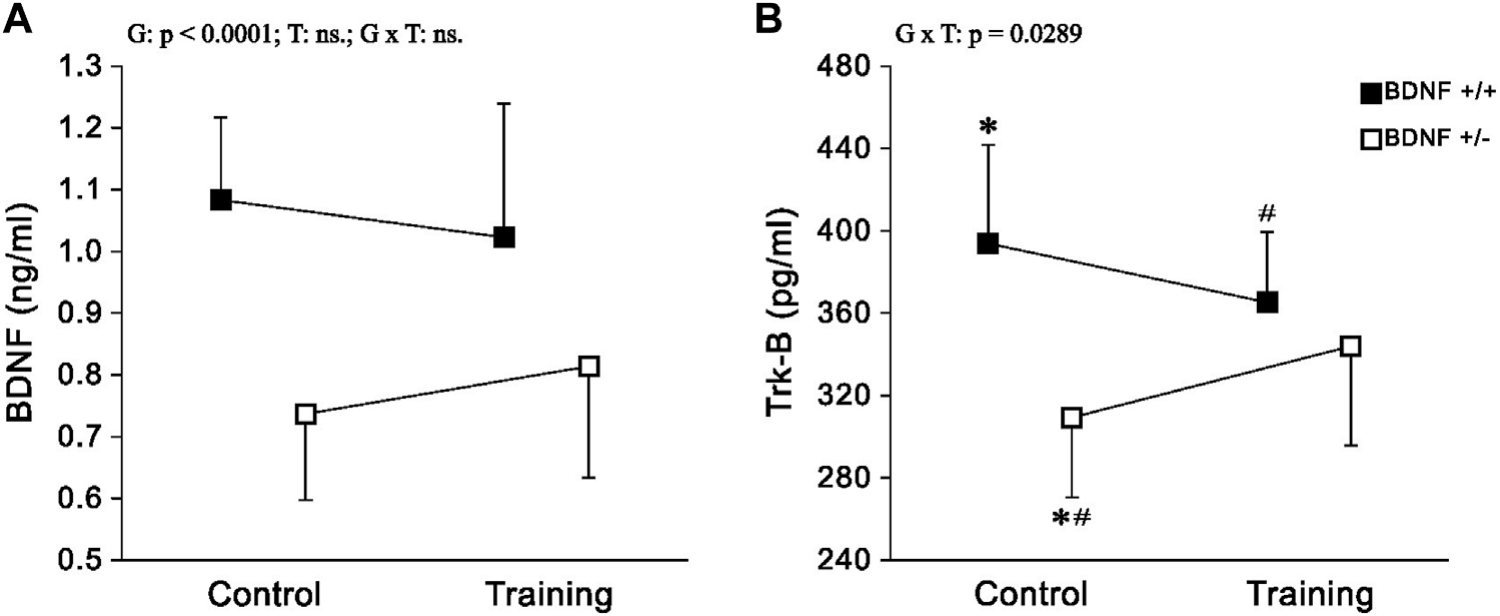
The mean values (±SD) of BDNF **(A)** and Trk-B **(B)** concentrations in the liver of rats from control (left side) and trained (right side) groups. Control and trained *Bdnf*+/+ groups are indicated by black square, while control and trained *Bdnf*+/− groups are indicated by white square. Comparisons between groups were made by two-way ANOVA. (G) genotype (*Bdnf*+/+ genotype vs. *Bdnf*+/− genotype); T—training (control vs. training); G x T, the interaction between genotype and training. For significant interactions Tukey’s *post hoc* test was performed. Post-hoc results: * = significant difference between control rats with *Bdnf*+/+ genotype and control rats with *Bdnf*+/− genotype (*p* = 0.0006); # = significant difference between trained rats with *Bdnf*+/+ genotype and control rats with *Bdnf*+/− genotype (*p* = 0.0351). Detailed results of performed tests are given in Table 1.

*BDNF and Trk-B* There was a statistically significant reduction in liver levels of both BDNF and Trk-B in rats from the knockout population (*Bdnf*+/− and *Bdnf*+/−T) when compared to rats from normal genotype populations (*Bdnf*+/+ and *Bdnf*+/+T) (Figures 2A, B; Table 1). Significantly lower level of Trk-B was found in *Bdnf*+/− group of rats in relation to either *Bdnf*+/+ or *Bdnf*+/+T groups of rats (Figure 2B; Table 1). However, no changes in hepatic BDNF and TrkB were found when populations of control (*Bdnf*+/+ *and Bdnf*+/−) and trained (*Bdnf*+/+T *and Bdnf*+/−T) rats were compared (Figures 2A,B; Table 1).

**TABLE 1:** Results of significance (p), effect size (ηp2) and test power (F) for particular effects and interactions regarding studied biomarkers such as: BDNF and Trk-B (Figures 2A,B), lipids–CHOL, LDL and TG (Figures 3A–C), enzymes—ALAT, ASAT, ALP, GGT, LDH (Figures 4A–E), hormones–INS, LEP (Figures 5A,B) and IL-6 (Figure 6A). Two-way analysis of variance (ANOVA) was performed. Genotype (*Bdnf*+/+ genotype vs. *Bdnf*+/− genotype); Training (control group vs. trained group); Genotype/Training (the interaction between genotype and training).

Studied biomarkers		Genotype	Training	Genotype/Training
**BDNF/TrkB signaling**				
BDNF	F_1,37_	27.45	0.03	1.68
	p	<0.0001	0.8737	0.2027
	η*p* ^2^	0.43	0	0.04
Trk-B	F_1,34_	14.59	0.05	5.21
	p	0.0005	0.829	0.0289
	η*p* ^2^	0.3	0	0.13
**Lipids**				
CHOL	F_1,34_	27.4	0	0.17
	p	<0.0001	0.9593	0.6793
	η*p* ^2^	0.45	0	0.01
LDL	F_1,37_	35.11	3.18	1.3
	p	<0.0001	0.0826	0.2611
	η*p* ^2^	0.49	0.08	0.03
TG	F_1,33_	14.56	3.85	0.32
	p	0.0006	0.0582	0.5772
	η*p* ^2^	0.31	0.1	0.01
**Enzymes**				
LDH	F_1,34_	0.01	60.1	1.43
	p	0.9149	<0.0001	0.2397
	η*p* ^2^	0	0.64	0.04
ALAT	F_1,32_	15.44	3.13	5.26
	p	0.0004	0.0862	0.0285
	η*p* ^2^	0.33	0.09	0.14
ASAT	F_1,33_	1.26	9.74	2.40
	p	0.2700	0.0037	0.1307
	η*p* ^2^	0.04	0.23	0.07
GGT	F_1,36_	59.88	3.43	0
	p	<0.0001	0.0724	0.9718
	η*p* ^2^	0.62	0.09	0
ALP	F_1,33_	0	1.56	0.14
	p	0.981	0.2211	0.7152
	η*p* ^2^	0	0.05	0
**Hormones**				
INS	F_1,34_	0.48	25.16	1.2
	p	0.4936	<0.0001	0.2819
	η*p* ^2^	0.01	0.43	0.03
LEPT	F_1,33_	0.69	21.22	2.98
	p	0.4131	<0.0001	0.0938
	η*p* ^2^	0.02	0.39	0.08
**Interleukin**				
IL-6	F_1,36_	5.48	3.24	4.98
	p	0.0249	0.0801	0.032
	η*p* ^2^	0.13	0.08	0.12

**FIGURE 3:**
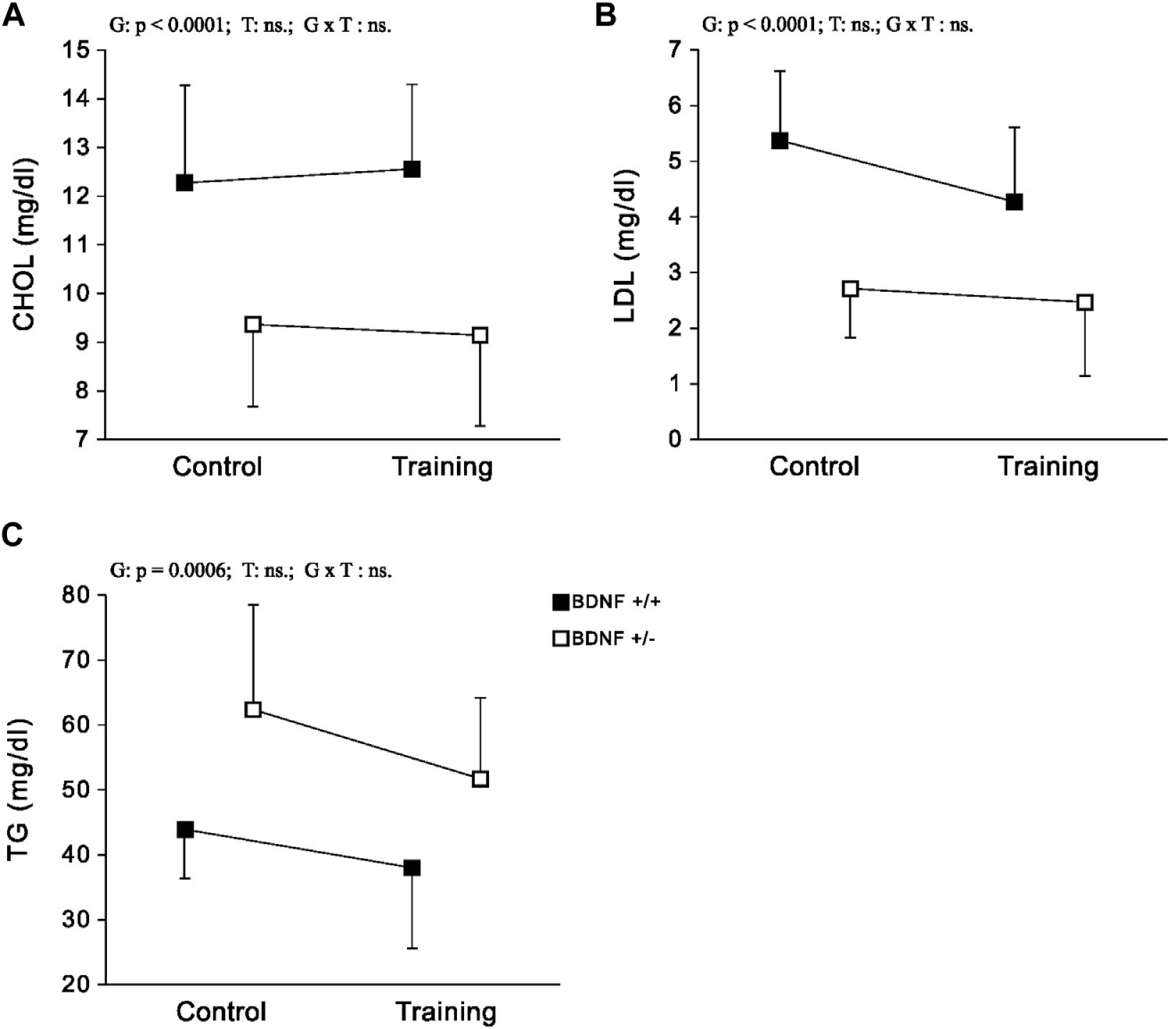
The mean values (±SD) of CHOL **(A)**, LDL **(B)** and TG **(C)** concentrations in the liver of rats from control (left side) and trained (right side) groups. Control and trained *Bdnf*+/+ groups are indicated by black square, while control and trained *Bdnf*+/− groups are indicated by white square. Comparisons between groups were made by two-way ANOVA. (G) genotype (*Bdnf*+/+ genotype vs. *Bdnf*+/− genotype); T—training (control vs. training); G x T, the interaction between genotype and training. Significant results are denoted by p, while non-significant results are denoted by ns. Detailed results of performed tests are given in Table 1.

*Lipids* CHOL and LDL levels were significantly lower, (but significantly higher for TG) in the population of knockout rats (*Bdnf*+/− and *Bdnf*+/−T) compared to the population of rats with typical genotype (*Bdnf*+/+ and *Bdnf*+/+T) (Figure 3; Table 1). Endurance training did not change the liver contents of CHOL, LDL and TG. Their concentrations were similar in control (*Bdnf+/+* and *Bdnf+/*−) and trained (*Bdnf+/+*T and *Bdnf+/*−T) populations (Figure 3; Table 1). However, there was a clear downward trend in TG concentration in trained rats (*Bdnf*+/+T and *Bdnf*+/−T).

**FIGURE 4:**
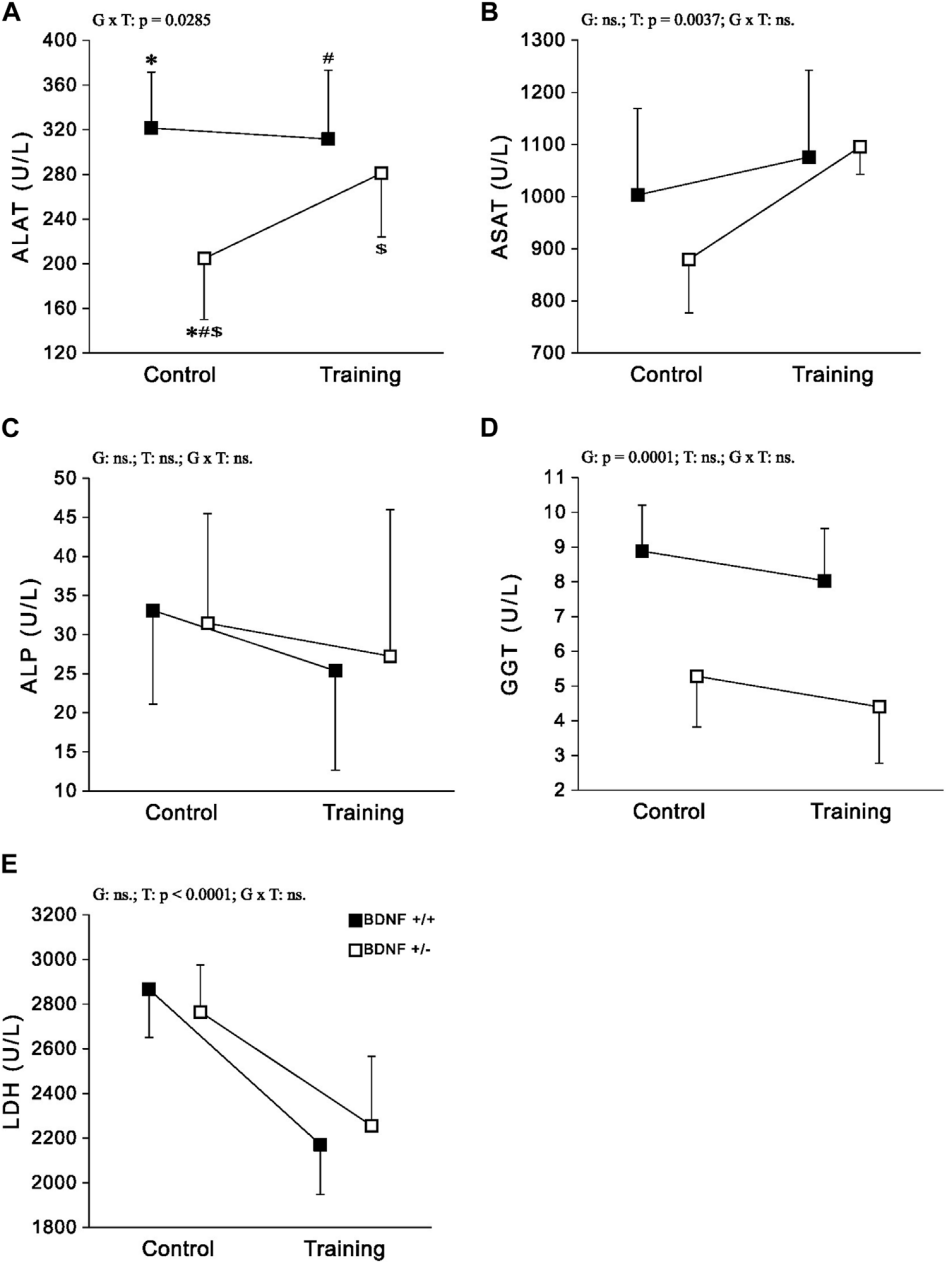
The mean values (±SD) of ALAT **(A)**, ASAT **(B)**, ALP **(C)**, GGT **(D)** and LDH **(E)** activities in the liver of rats from control (left side) and trained (right side) groups. Control and trained *Bdnf*+/+ groups are indicated by black square, while control and trained *Bdnf*+/− groups are indicated by white square. Comparisons between groups were made by two-way ANOVA. (G) genotype (*Bdnf*+/+ genotype vs. *Bdnf*+/− genotype); T—training (control vs. training); G x T, the interaction between genotype and training. significant results are denoted by p, while non-significant results are denoted by ns. For significant interactions Tukey’s *post hoc* test was performed. Post-hoc results: * = significant difference between control rats with *Bdnf*+/+ genotype and control rats with *Bdnf*+/− genotype (*p* = 0.0003); # = significant difference between trained rats with *Bdnf*+/+ genotype and control rats with *Bdnf*+/− genotype (*p* = 0.0014); $ = significant difference between trained rats with *Bdnf*+/− genotype and control rats with *Bdnf*+/− (*p* = 0.0366). Detailed results of performed tests are given in Table 1.

*Enzymes* Significantly lower activities of ALAT and GGT were observed in the population of knockout rats (*Bdnf*+/− and *Bdnf*+/−T) compared to the corresponding population of normal genotype rats (*Bdnf*+/+ and *Bdnf*+/+T) (Figures 4A,D; Table 1). However, ALAT activity was lower in the control group of knockout rats compared to the other groups (Figures 4B,C,E; Table 1). In contrast, there was no statistically significant difference in hepatic ASAT, LDH, and ALP activities between normal genotype rats (*Bdnf*+/+ and *Bdnf*+/+T) and knockout genotype rats (*Bdnf*+/− and *Bdnf*+/−T). There were no differences in hepatic ALP and GGT activities between the control (*Bdnf+/+* and *Bdnf+/*−) and trained (*Bdnf+/+*T and *Bdnf+/*−T) populations of rats (Figures 4A,C,D; Table 1). On the other hand, there was increase in both ALAT (not significant) and ASAT activities (Figure 2B; Table 1) as well a statistically significant decrease in LDH activity (Figure 4E; Table 1) in the liver of trained (*Bdnf+/+*T and *Bdnf+/*−T) in compare to control rat population (*Bdnf+/+* and *Bdnf+/*−). However, this post-training elevation of ALAT and ASAT activities resulted from an increase of these enzymes activities the only in trained group of rats with BDNF knockout genotype (Figures 4A,B; Table 1).

**FIGURE 5:**
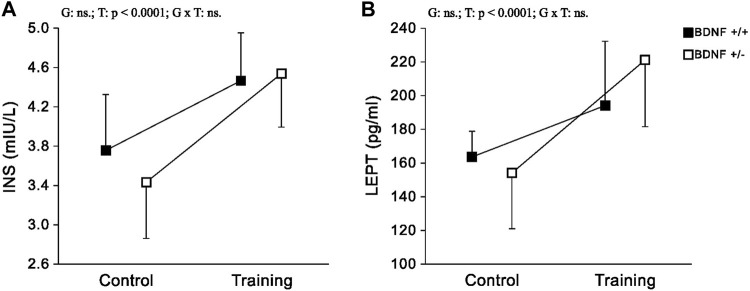
The mean values (±SD) of INS **(A)** and LEPT **(B)** concentrations in the liver of rats from control (left side) and trained (right side) groups. Control and trained *Bdnf*+/+ groups are indicated by black square, while control and trained *Bdnf*+/− groups are indicated by white square. Comparisons between groups were made by two-way ANOVA. (G) genotype (*Bdnf*+/+ genotype vs. *Bdnf*+/− genotype); T - training (control vs. training); G x T, the interaction between genotype and training. Significant results are denoted by p, while non-significant results are denoted by ns. Detailed results of performed tests are given in Table 1.

*Hormones* The concentrations and activities of INS and LEPT in the liver did not differ when the population of typical genotype rats (*Bdnf*+/+ and *Bdnf*+/+T) was compared with the corresponding population of knockout genotype rats *Bdnf*+/− *Bdnf*+/−T) (Figure 5; Table 1). Liver concentrations of INS and LEPT were significantly higher in the population of trained rats *(Bdnf+/+T* and *Bdnf+/*−*T)* compared to the corresponding population of control rats *(Bdnf+/+* and *Bdnf+/*−*)* (Figure 5; Table 1).

**FIGURE 6:**
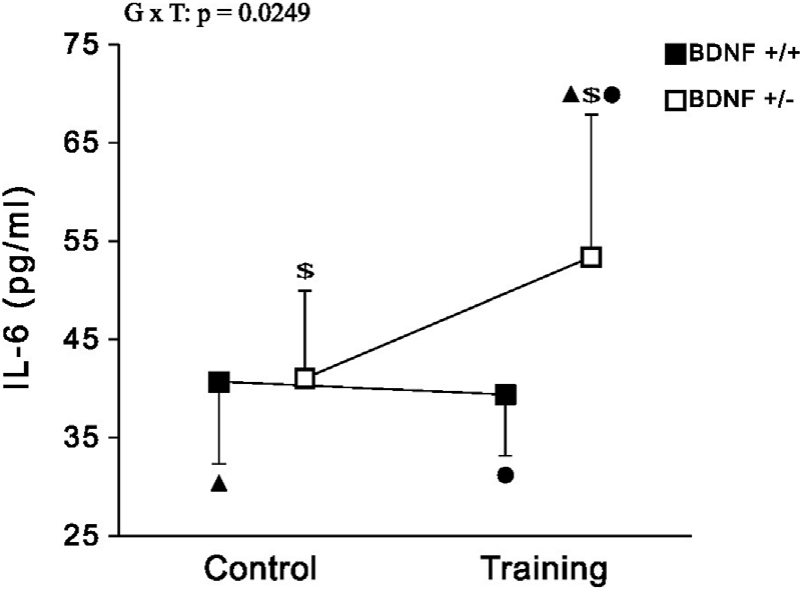
The mean values (±SD) of IL-6 (A) concentration in the liver of rats from control (left side) and trained (right side) groups. Control and trained *Bdnf*+/+ groups are indicated by black square, while control and trained *Bdnf*+/− groups are indicated by white square. Comparisons between groups were made by two-way ANOVA. G—genotype (*Bdnf*+/+ genotype vs. *Bdnf*+/− genotype); T - training (control vs. training); G x T, the interaction between genotype and training. For significant interactions Tukey’s *post hoc* test was performed. Post-hoc results: ▲ = significant difference between trained rats with Bdnf+/− genotype and control rats with *Bdnf*+/+ genotype (*p* = 0.0354); ● = significant difference between trained rats with *Bdnf*+/− genotype and trained rats with *Bdnf*+/+ genotype (*p* = 0.0201); $ = significant difference between trained rats with *Bdnf*+/− genotype and control rats with Bdnf+/− (*p* = 0.0422). Detailed results of performed tests are given in Table 1.

*IL-6* The concentration of IL-6 in the liver of rats from the knockout population (*Bdnf*+/− and *Bdnf*+/−T) was demonstrated to be statistically higher than that of rats from the normal genotype population (*Bdnf*+/+ and *Bdnf*+/+T) (Figure 6; Table 1). However, this difference in IL-6 levels shown between rat populations with different genotypes was caused by a significant increase in IL-6 concentration in the group of trained animals with BDNF gene knockout (see Figure 6 and Table 1) Comparing IL-6 levels in the liver between the control (*Bdnf*+/+ and *Bdnf*+/−) and trained (*Bdnf*+/+T and *Bdnf*+/−T) populations, no significant difference in IL-6 levels was observed. However, the *Bdnf*+/−T group showed a significant increase in IL-6 levels compared to the other groups (Figure 6; Table 1).

## Discussion

In the current study, there was an increase in body and liver weight, as well as a decrease in liver BDNF and Trk-B concentrations in BDNF knockout rats (*Bdnf+/*− and *Bdnf*+/−T) compared to normal genotype rats (*Bdnf*+/+ and *Bdnf*+/+T) (Figures 1A,B; Table 1). Moreover, our previous research (Grzelak et al., 2023) demonstrated that BDNF knockout rats had significantly lower serum BDNF levels than rats with normal genotype. Taken together, these results showed that rats with hemizygous deletion of the BDNF gene exhibited reduced serum BDNF levels and impaired hepatic BDNF/TrkB signaling in the liver. These both findings provide evidence to support the use of these rats as a genetic model to study metabolic syndrome. Moreover, this seems to be consistent with the report by Belviranli and Okudan (2018), which suggests a strong connection between BDNF levels in the liver and circulating blood.

Additionally, our study indicated that reduced BDNF and Trk-B signaling in the liver, resulting from the absence of one BDNF allele, has a particularly important impact on lipids metabolism in hepatocytes. Differences in lipid concentrations, including CHOL, LDL and TG, were evident when comparing the knockout with normal genotype rat populations. In particular, hepatic CHOL and LDL levels were significantly reduced in BDNF knockout rats (*Bdnf*+/− and *Bdnf*+/−T) compared to those with normal genotypes (*Bdnf*+/+ and *Bdnf*+/+T). CHOL is synthesised directly by hepatocytes in the liver (Zhou and Sun, 2021), while LDL is formed in the liver from particles of very low-density lipoproteins (Feingold, 2022). LDL plays a key role in transporting cholesterol from the liver to peripheral cells, according to previous research (Eckardstein, 2020). Moreover, several studies have demonstrated an inverse relationship between the concentration of CHOL (Abdel-Gayoum and Ahmida, 2017) and LDL (Ogunmoyole et al., 2019) in the liver and their corresponding levels in serum. Thus, our results suggest that CHOL accumulation and storage in the liver may be impaired in rats with hemizygous BDNF deletion. This also indicates that knockout rats may be subject to CHOL accumulation in the arteries, which may lead to the formation of atherosclerotic plaques—a major contributor to atherosclerosis (Subczynski and Pasenkiewicz-Gierula, 2020). It is therefore reasonable that knockout rats may have an increased risk of developing cardiovascular. Such a suggestion is in line with previous human studies that have linked reduced serum or plasma BDNF levels to a higher incidence of various cardiovascular diseases (Chaldakov et al., 2004; Sustar et al., 2019).

We also found that liver TGs levels were significantly higher in knockout rats compared to normal genotype rats. TGs are obtained from dietary sources as well as synthesised in the liver as part of the process of lipid metabolism, where they are stored in hepatocytes (Alves-Bezerra and Cohen, 2017). Elevated levels of TGs in the liver caused by increase food intake, as observed in knockout rats, can lead to increase liver weight and eventually induce fatty liver (Garnol et al., 2016). This may explain the considerable increase in liver weight observed in knockout rats. Furthermore, it can be supposed that elevated hepatic TGs levels may be responsible for increased body weight in these rats, as excess TGs are stored in adipose tissue, leading to obesity (Ahmadian et al., 2009). Based on all the aforementioned observations, it can be strongly inferred that the significant reduction in BDNF/Trk-B signaling in the liver, observed in genetically modified rats, plays a key role in inducing substantial changes in hepatic lipid metabolism. This finding is also supported by the study conducted by Tsuchida et al. (2002), which demonstrated that subcutaneous administration of BDNF could lead to significant reductions in liver weight, TG concentrations, and the development of fatty liver in obese diabetic mice. Therefore, it can be concluded that a decrease in BDNF/Trk-B signaling in the liver is a crucial factor that contributes to the onset of metabolic syndrome symptoms.

The moderate-intensity endurance training applied in our research did not alter either the BDNF and Trk-B content (Figure 1 A and B; Table 1) or the CHOL and LDL concentrations in the liver of the studied rats. Long-term aerobic exercise, particularly at high intensity, is recommended as an effective therapy for improving lipid profiles (Mann et al., 2014) or to counteract metabolic syndrome (Mrówczyński, 2019). However, 5-weeks endurance training of graduate increasing intensity, utilized in the present study, did not result in elevated levels of BDNF and Trk-B in the liver, suggesting that it may have been insufficient to induce significant improvement in the lipid profile. It is important to emphasise that our previous research has shown that training at such intensity can lead to an increase in both BDNF and Trk-B levels in some fast hindlimb muscles (Grzelak et al., 2023). However, there is no a significant effect on circulating levels of BDNF what seems to be in line with results of Gaitan et al. (2021) who found no increase of BDNF level in the blood of adult humans even after 26 weeks of aerobic exercise. However, it cannot be ruled out that the implementation of high-intensity or prolonged endurance activity may evoke increase in BDNF levels in both the serum and the liver which could improve of the lipid profile. It has been reported that high-intensity interval training has a particularly desirable effect on lipid metabolism by reducing their concentrations in the blood, including levels of CHOL, TGs and others (Cao et al., 2022).

Knockout rats (*Bdnf*+/− and *Bdnf*+/−T) showed a decrease in liver metabolic enzyme activities, specifically ALAT and GGT compared to rats with a normal genotype (*Bdnf*+/+ and *Bdnf*+/+T). ALAT is a specific liver enzyme crucial for amino acid metabolism (Glinghammar et al., 2009) while GGT is a key enzyme in the metabolism of glutathione (Hanigan et al., 2015). Higher activities of these enzymes in the liver indicate more efficient enzyme function, as their levels in the liver are inversely proportional to those in the blood. However, liver disorders can cause the release of these enzymes from hepatocyte cytoplasm into the blood, where they serve as indicators of impaired metabolic function in liver disorders (Vermeulen et al., 1992; Deoras et al., 1997; Ogunmoyole et al., 2019). ALAT and GGT levels are routinely measured in serum or plasma to investigate liver disease (Green and Flamm, 2002; Carobene et al., 2013) due to these both markers are particularly relevant in the context of pathogenesis of various liver diseases related to fat accumulation in liver such as obesity, metabolic syndrome and the cardiovascular disease (Sookoian and Pirola, 2015). For example, elevated levels of these markers have been observed in children as a consequence of liver damage associated with overweight and obesity (Johansen et al., 2020). Therefore, it is reasonable to speculate that knockout rats would exhibit increased activities of ALAT and GGT in circulation, indicating disruptions in metabolic processes within the liver.

Our results demonstrated that endurance training led to an increase in hepatic ALAT activity and a significant elevation in ASAT activity in knockout rats (*Bdnf*+/− and *Bdnf*+/−T). However, this effect was mainly observed in the group of trained knockout rats (Figures 5B,C,E; Table 1). It is well-established that endurance training can improve liver function and reduce liver enzyme levels in the bloodstream, such as ALAT and ASAT, particularly in individuals with obesity or metabolic syndrome (Haukeland et al., 2008; Skrypnik et al., 2016). Therefore, our findings demonstrate an increase in ALAT and ASAT levels in the liver of knockout rats, confirmed previous observations. The implemented training regimen led to a significant reduction in hepatic LDH activity in the trained rat population (*Bdnf*+/+T and *Bdnf*+/−T), an enzyme crucial for cellular metabolism, particularly anaerobic glycolysis (Farhana and Lappin, 2023). Studies by Lupa et al. (1994), have previously demonstrated a decrease in hepatic LDH activity in response to endurance training in young rats. This finding suggests that endurance training may have a positive impact on liver health and metabolic function, potentially reducing the risk of liver-related disorders. Importantly, our results indicate that the decrease in hepatic LDH activity observed after endurance training is independent of the BDNF genotype.

No significant differences were observed in INS and LEPT hormone concentrations in the livers of rats with knockout (*Bdnf*+/− and *Bdnf*+/−T) and normal (*Bdnf*+/+ and *Bdnf*+/+T) genotypes. These findings indicate that liver functions, including the regulation of glucose metabolism (Chadt and Al-Hasani, 2020) and maintenance of energy balance (Martínez-Uña et al., 2020) remain unchanged in knockout rats. This can be attributed to the fact that this liver function is closely related to hormones produced by other organs and transported to the liver via the circulation. However, it is worth noting that liver INS and LEPT levels were significantly higher after endurance training in our study. This observation is therefore consistent with previous reports emphasising the importance of regular exercise in regulating body weight and blood sugar levels in humans (Karacabey, 2009). Interestingly, our results indicate that these training effects are not dependent on BDNF genotypes, as observed for LDH activity.

It is important to note that a significant reduction in BDNF and Trk-B levels in liver of knockout rats (*Bdnf*+/− and *Bdnf*+/−T) seems to be also associated with a higher concentration of hepatic IL-6 compared to rats with the normal genotype (*Bdnf*+/+ and *Bdnf*+/+T). However, such difference is caused by significant increase in IL-6 concentration in the group of trained animals with BDNF gene deficiency (see Figure 2 and Table 1). Cressman et al. (1996) discovered that even a single dose of IL-6 can restore gene expression in mice lacking IL-6 gene, resulting in hepatocyte proliferation and inhibition of liver damage. Garnol et al. (2016) observed heightened levels of IL-6 in the liver after partial hepatectomy, considering it as an essential factor for initiating liver regeneration. Therefore, elevated levels of IL-6 play a crucial role in promoting hepatocyte proliferation and are commonly regarded as a key factor in driving liver regeneration. Accordingly, our findings have demonstrated a significant increase in hepatic IL-6 levels, particularly in the trained knockout rat group (Figure 2; Table 1). This suggests that endurance training has the potential to enhance hepatocyte proliferation and initiate liver regeneration processes, especially in animals with reduced levels of BDNF and Trk-B in the liver. Additionally, it is well-established that muscle cells are a major source of IL-6 production in response to exercise. Steinbacher and Eckl (2015) proposed that IL-6 produced by muscles during exercise exerts an anti-inflammatory effect, leading to a reduction in pro-inflammatory cytokine levels. Moreover, it has been observed that muscles release a substantial amount of IL-6 into the bloodstream even after a single exercise session (Fischer, 2006), and this IL-6 can be captured by the liver (Febbraio et al., 2003). Therefore, our results suggest that the production of IL-6 during training plays a crucial role in promoting liver regeneration, particularly in animals with metabolic liver disorders like knockout rats. Furthermore, the elevated IL-6 levels in BDNF-deficient rats may be a potential factor contributing to the increase in ALAT and ASAT activity. This suggests that IL-6 might play a regenerative role by enhancing the efficiency of some metabolic pathways.

## Conclusion

The study revealed that BDNF knockout rats loss exhibited a significant reduction in BDNF/Trk-B content within the liver. This reduction was accompanied by lower levels of lipids, such as CHOL and LDL, and a simultaneous increase in TGs levels. In addition, there was a reduction in the activity of two liver enzymes—ALAT and GGT. These alterations in lipid metabolism and enzyme activity contributed to increase in obesity, as evidenced by elevated liver weight and body weight in the rats. Our findings suggest that a reduction in BDNF/Trk-B signaling contributes to changes in lipid metabolism that lead to hepatic steatosis and obesity, which are symptoms of the metabolic syndrome.

Moderate endurance training induced an increase in hepatic ALAT and ASAT activity in knockout rats, suggesting improved metabolic pathway efficiency in the liver. Furthermore, a significant increase in hepatic IL-6 levels was demonstrated in trained animals with the BDNF knockout genotype, suggesting that the initiation of liver regeneration is mediated by IL-6 produced by contracting skeletal muscle. It cannot be excluded that it is responsible for improving the efficiency of metabolic pathways by increasing ALAT and ASAT activity. On the other hand, the effect of endurance exercise on LEPT and INS concentrations, as well as LDH activity, was not dependent on a specific BDNF genotype.

Future studies should focus on investigating the effects of the BDNF/Trk-B axis on the pathways of the above-mentioned enzymes, which play a key role in liver metabolic functions. Furthermore, upcoming studies should aim to provide a more complex analysis of the role of IL-6 in liver regeneration.

## Limitations

Measurement of the biomarkers tested in blood was not possible due to the fact that blood samples were collected to assess neurotrophin levels, as shown in our previous work performed on the same group of animals (Grzelak et al., 2023). The volume of blood samples collected was also limited due to the *in vivo* electrophysiology experiment being conducted in parallel. In addition, we did not conducted immunochemistry research in liver.

## Data Availability

The original contributions presented in the study are included in the article/Supplementary Material, further inquiries can be directed to the corresponding author.
